# Hypoperfused tissue salvage after selective intra-arterial hypothermia during mechanical thrombectomy for anterior circulation large-vessel occlusion

**DOI:** 10.3389/fneur.2026.1890819

**Published:** 2026-08-12

**Authors:** QianQian Bi, YongPeng Wang, Wang Fu, Qiwei Wang, WenHao Yang, JingYu Gu, SiYang Ren, QuanBin Zhang, Feng Wang

**Affiliations:** 1 Department of Neurology, Seventh People's Hospital of Shanghai University of Traditional Chinese Medicine, Shanghai, China; 2 Department of Radiology, Seventh People's Hospital of Shanghai University of Traditional Chinese Medicine, Shanghai, China; 3 Department of Neurosurgery, Shanghai Tenth People's Hospital, Tongji University School of Medicine, Shanghai, China

**Keywords:** acute ischemic stroke, hypoperfused tissue salvage volume, mechanical thrombectomy, neuroprotection, selective intra-arterial hypothermia

## Abstract

**Objectives:**

To evaluate whether selective intra-arterial hypothermia during mechanical thrombectomy is associated with greater preservation of initially hypoperfused tissue in patients with anterior-circulation large-vessel occlusion.

**Methods:**

This prospective single-center nonrandomized comparative cohort study included consecutive patients with anterior-circulation large-vessel occlusion treated with mechanical thrombectomy. Patients received selective intra-arterial hypothermia or standard treatment at the discretion of the treating interventionist after informed consent was obtained. The primary endpoint was hypoperfused tissue salvage volume, defined as baseline hypoperfusion volume on computed tomographic perfusion minus final infarct volume on follow-up magnetic resonance imaging. Secondary outcomes were analyzed descriptively without formal adjustment for multiple comparisons.

**Results:**

Ninety-four patients were included, with 45 in the hypothermia group and 49 in the standard-treatment group. The prespecified primary endpoint, hypoperfused tissue salvage volume, did not differ significantly between groups: median hypoperfused tissue salvage volume was 107.85 mL (interquartile range, 53.80–159.64) in the hypothermia group and 81.96 mL (interquartile range, 50.23–143.80) in the standard-treatment group (*p* = 0.384). In adjusted median regression, hypothermia was not significantly associated with tissue salvage volume (adjusted median difference, 21.08 mL; 95% confidence interval, −23.47 to 65.63; *p* = 0.350). Final infarct volume and safety outcomes did not differ significantly between groups. Descriptive secondary analyses showed nominally greater day-7 NIHSS improvement in the hypothermia group (6.2 ± 5.9 vs. 3.8 ± 5.7, *p* = 0.043), and a higher proportion of 90-day functional independence (82.2% vs. 61.2%, *p* = 0.039).

**Conclusion:**

Selective intra-arterial hypothermia during mechanical thrombectomy was not associated with a significant improvement in hypoperfused tissue salvage volume, the prespecified primary endpoint. Nominally favorable secondary clinical signals should be interpreted as exploratory and require confirmation in larger, adequately powered randomized trials.

## Introduction

Mechanical thrombectomy is the standard reperfusion therapy for acute ischemic stroke caused by large-vessel occlusion, yet successful recanalization does not invariably translate into tissue preservation or favorable functional recovery (1, 2). One explanation is that ischemic injury may continue to evolve despite reopening of the culprit artery because of microcirculatory no-reflow, blood–brain barrier disruption, oxidative stress, inflammatory activation, and reperfusion injury (3, 4).

Therapeutic hypothermia remains one of the most extensively studied neuroprotective strategies in experimental stroke. Cooling lowers metabolic demand and may attenuate excitotoxic, oxidative, and inflammatory injury; even small temperature differences can influence the extent of ischemic damage (4–6). Selective intra-arterial hypothermia is attractive in the thrombectomy setting because it allows cold saline to be delivered directly into the ischemic vascular territory while limiting systemic exposure (6–8).

Prior clinical work on selective intra-arterial hypothermia has focused mainly on feasibility, safety, and broad clinical outcomes rather than direct assessment of tissue preservation after reperfusion (7–12). In contrast, hypoperfused tissue salvage volume, calculated as baseline hypoperfusion volume minus final infarct volume, provides a tissue-level estimate of how much initially threatened tissue is ultimately preserved. This concept is aligned with perfusion-based definitions of tissue at risk used in modern thrombectomy imaging paradigms (13, 14).

We used hypoperfused tissue salvage volume as the primary endpoint in this prospective, single-center, nonrandomized comparative cohort. We hypothesized that selective intra-arterial hypothermia used as an adjunct to mechanical thrombectomy would be associated with greater preservation of initially hypoperfused tissue than standard thrombectomy alone.

## Methods

### Study design and setting

This study was a prospective single-center nonrandomized comparative cohort study conducted at the Seventh People’s Hospital of Shanghai University of Traditional Chinese Medicine. The study protocol was approved by the Seventh People’s Hospital of Shanghai University of Traditional Chinese Medicine Institutional Review Board (7th-HIRB-090). Written informed consent for treatment and data collection was obtained from each patient or a legally authorized representative according to institutional practice.

### Participants

Consecutive patients with acute ischemic stroke caused by anterior-circulation large-vessel occlusion and treated with mechanical thrombectomy were eligible for inclusion. Patients were required to be 18–90 years of age and to have a prestroke modified Rankin Scale (mRS) score of 0 or 1. Patients were excluded if they had intracranial hemorrhage on baseline imaging, intracranial tumor or central nervous system infection, severe coagulation abnormality, or severe heart failure judged to contraindicate the procedure.

### Treatment groups and procedures

After informed consent had been obtained, patients underwent selective intra-arterial hypothermia or standard treatment according to the treating interventionist’s real-world clinical judgment. All patients underwent mechanical thrombectomy according to the standard institutional workflow.

In the hypothermia group, once the microcatheter had crossed the thrombus and distal patency had been confirmed, 4 °C normal saline was infused through the microcatheter at 10 mL/min for 3 min before reperfusion, for a total prerecanalization cold saline volume of 30 mL. Recanalization was then performed using SOLUMBAR or SWIM techniques, with balloon angioplasty and/or stenting when necessary to maintain antegrade flow. Successful recanalization was defined as a final mTICI score of 2b–3. After completion of thrombectomy attempts, cold saline was further infused through the intermediate catheter at 30 mL/min for 10 min, for a post-thrombectomy volume of 300 mL. If successful recanalization was achieved, this infusion was administered after recanalization. If recanalization was unsuccessful or incomplete, defined as a final mTICI score <2b, and the operator decided to terminate further thrombectomy attempts, the infusion was still administered at the prespecified rate before catheter withdrawal. The total cold saline volume was therefore 330 mL. Patients in the standard-treatment group underwent conventional thrombectomy without cold saline perfusion. All other acute stroke management, including intravenous thrombolysis when indicated, followed routine institutional practice.

### Definitions and outcomes

Baseline computed tomographic perfusion maps were processed using RAPID software (version 5.0.4; iSchemaView, Menlo Park, CA, USA). Baseline ischemic core volume was defined as the preprocedural CT-perfusion volume with cerebral blood flow <30% of normal tissue, and baseline hypoperfusion volume was defined as the volume with Tmax >6.0 s (13, 14).

Final infarct volume was calculated based on MRI performed within 24–48 h after the procedure and was manually segmented using Horos software (version 3.3.6). Two clinicians independently outlined the infarct borders on each slice, with the total volume obtained by summing the individual slice volumes, and the average of the two measurements was used for analysis. Complete blinding of the imaging assessors to treatment allocation could not be guaranteed for all measurements, and formal inter-rater reliability analysis was not performed. The 90-day mRS and NIHSS assessments were performed during follow-up by dedicated clinicians who were blinded to treatment allocation.

The primary endpoint was hypoperfused tissue salvage volume, defined as baseline hypoperfusion volume on computed tomographic perfusion minus final infarct volume on follow-up MRI. This derived imaging variable was used to estimate the volume of initially hypoperfused tissue that did not progress to infarction. Negative values were retained in the primary analysis because baseline hypoperfusion volume and follow-up infarct volume were derived from different imaging modalities and time points, and negative values may reflect measurement variability or biological mismatch between initial perfusion abnormality and final infarction.

Key secondary efficacy endpoints were final infarct volume, 90-day mRS distribution, functional independence at 90 days (mRS 0–2), excellent functional outcome at 90 days (mRS 0–1), poor functional outcome at 90 days (mRS 4–6), early neurological improvement at day 7, and absolute NIHSS improvement from baseline to day 1, day 3, and day 7. Early neurological improvement was defined as a decrease of at least 4 NIHSS points from baseline or a day-7 NIHSS score of 0 or 1. Safety endpoints were any intracranial hemorrhage on follow-up imaging, intracranial vasospasm, in-hospital death, pneumonia, urinary tract infection, early neurological deterioration within 24 h, and 90-day mortality. Early neurological deterioration was defined as an increase of ≥4 points in the total NIHSS score from baseline within 24 h after treatment.

### Statistical analysis

Given the exploratory nature of this prospective single-center nonrandomized comparative cohort study, no formal sample-size or statistical power calculation was performed before study initiation, and consecutive eligible patients were enrolled during the study period. The findings were therefore interpreted as exploratory and require confirmation in larger adequately powered studies.

Continuous variables were summarized as mean ± standard deviation when approximately normally distributed and as median and interquartile range otherwise. Categorical variables were summarized as counts and percentages. Between-group comparisons used the Student *t* test or Mann–Whitney *U* test for continuous variables and the chi-square test or Fisher exact test for categorical variables, as appropriate.

Because hypoperfused tissue salvage volume was skewed and included occasional negative values, the primary unadjusted analysis compared tissue salvage volume between groups using the Mann–Whitney *U* test. The adjusted analysis used median regression with treatment group as the main explanatory variable and age, baseline NIHSS, intravenous thrombolysis, and onset-to-puncture time as covariates. Final infarct volume and NIHSS improvement were analyzed descriptively and with between-group comparisons using the prespecified methods described above. Ninety-day functional independence was explored using modified Poisson regression with robust variance estimation to generate adjusted risk ratios. Binary safety outcomes with low event counts were compared using the Fisher exact test.

As a sensitivity analysis for the primary endpoint, negative hypoperfused tissue salvage values were truncated at zero and compared between groups. Infarct growth was also analyzed as an alternative imaging metric. No formal adjustment for multiple comparisons was applied to secondary efficacy or safety outcomes. Therefore, *p* values for secondary outcomes were interpreted as nominal only, and all secondary analyses were considered exploratory and hypothesis-generating.

## Results

### Study population

**Figure 1:**
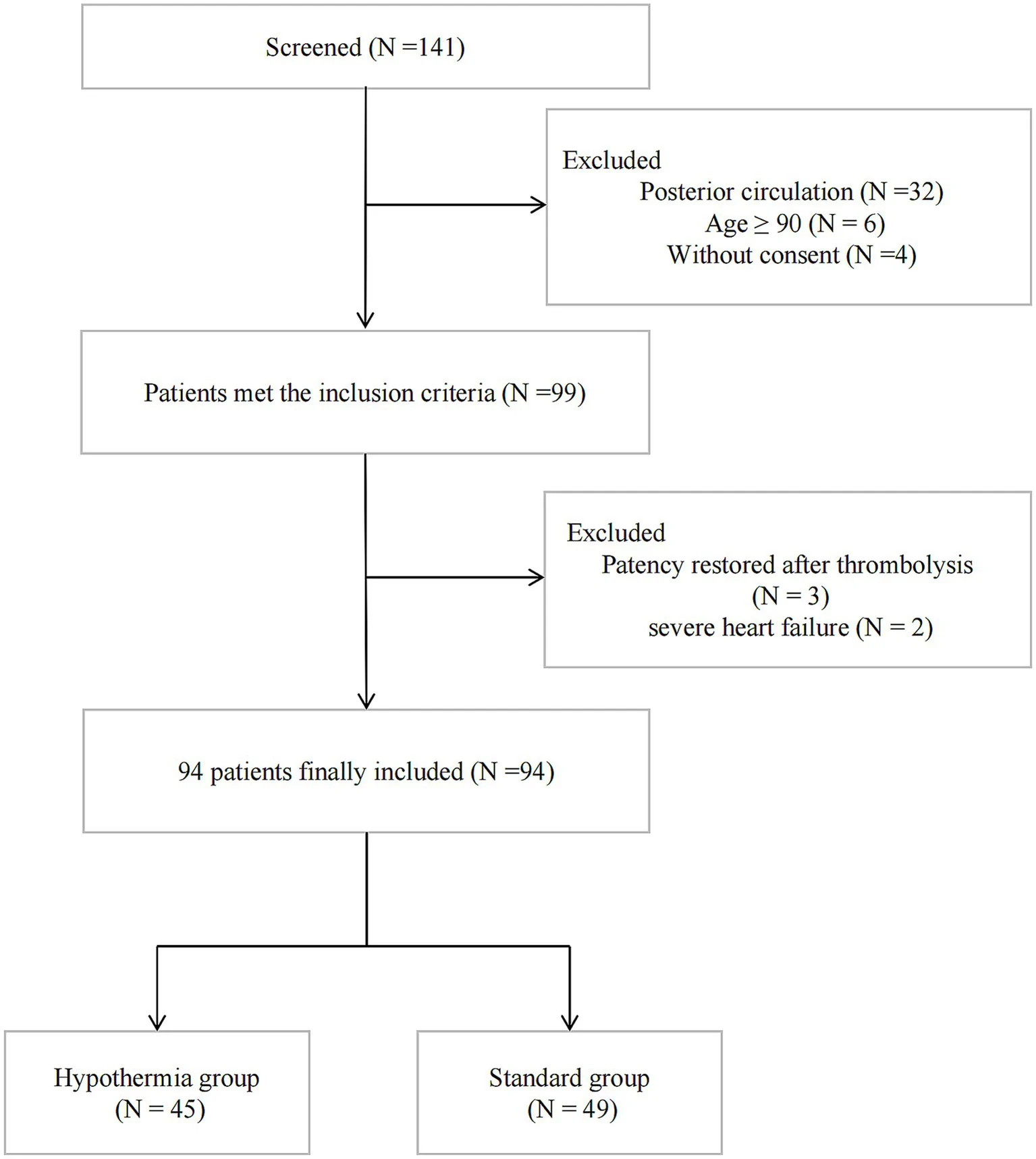
Screening flowchart.

A total of 94 patients were included in the analysis, comprising 45 patients in the selective intra-arterial hypothermia group and 49 patients in the standard-treatment group (Figure 1). The hypothermia group was somewhat younger than the standard-treatment group (mean age, 62.5 ± 10.7 vs. 66.5 ± 14.3 years), whereas baseline stroke severity was similar (median NIHSS, 8.0 [IQR, 5.0–14.0] vs. 7.0 [IQR, 5.0–12.0]). Intravenous thrombolysis was administered in 33.3 and 49.0% of patients, respectively. Baseline ischemic core volume and baseline hypoperfusion volume were comparable between groups (Table 1).

**Table 1:** Baseline characteristics.

Characteristic	Hypothermia group (*n* = 45)	Standard group (*n* = 49)	*p* value
Age, y	62.5 ± 10.7	66.5 ± 14.3	0.133
Male sex, *n*, %	33 (73.3%)	31 (63.3%)	0.377
Current smoking, *n*, %	9 (20.0%)	16 (32.7%)	0.243
Hypertension, *n*, %	31 (68.9%)	37 (75.5%)	0.498
Diabetes mellitus, *n*, %	16 (35.6%)	15 (30.6%)	0.664
Dyslipidemia, *n*, %	5 (11.1%)	13 (26.5%)	0.070
Prior stroke, *n*, %	8 (17.8%)	10 (20.4%)	0.798
Intravenous thrombolysis, *n*, %	15 (33.3%)	24 (49.0%)	0.146
Left-sided lesion, *n*, %	22 (48.9%)	17 (34.7%)	0.210
Occlusion site, *n*, %			0.058
Intracranial ICA	7 (15.6%)	14 (28.6%)	
Middle cerebral artery	38 (84.4%)	32 (65.3%)	
Anterior cerebral artery	0 (0.0%)	3 (6.1%)	
Onset to puncture, min	437.0 (229.0–709.0)	320.0 (215.0–614.0)	0.348
Door to puncture, min	116.0 (109.0–138.0)	117.0 (91.0–138.0)	0.364
Baseline NIHSS, median (IQR)	8.0 (5.0–14.0)	7.0 (5.0–12.0)	0.735
mTICI^*^, *n*, %			0.907
0	1 (2.2%)	1 (2.0%)	
1	0 (0%)	1 (2.0%)	
2a	1 (2.2%)	1 (2.0%)	
2b	13 (28.9%)	11 (22.4%)	
3	30 (66.7%)	35 (71.4%)	
Baseline ischemic core^*^, mL, median (IQR)	2.3 (0.1–8.9)	2.3 (0.0–12.9)	0.887
Baseline hypoperfusion volume^**^, mL, median (IQR)	116.3 (68.9–176.4)	102.3 (69.0–192.0)	0.565

Continuous variables are shown as mean ± SD or median (IQR); binary variables are shown as *n* (%). Low-missing continuous variables were MI-completed for display.

^*^Baseline ischemic core was defined as cerebral blood flow of less than 30% of that in normal tissue on computed tomographic perfusion imaging before mechanical thrombectomy.

^**^Baseline hypoperfusion volume was defined as one with a time to maximum (Tmax) delay of more than 6 s on computed tomographic perfusion imaging before mechanical thrombectomy.

### Primary endpoint

**Figure 2:**
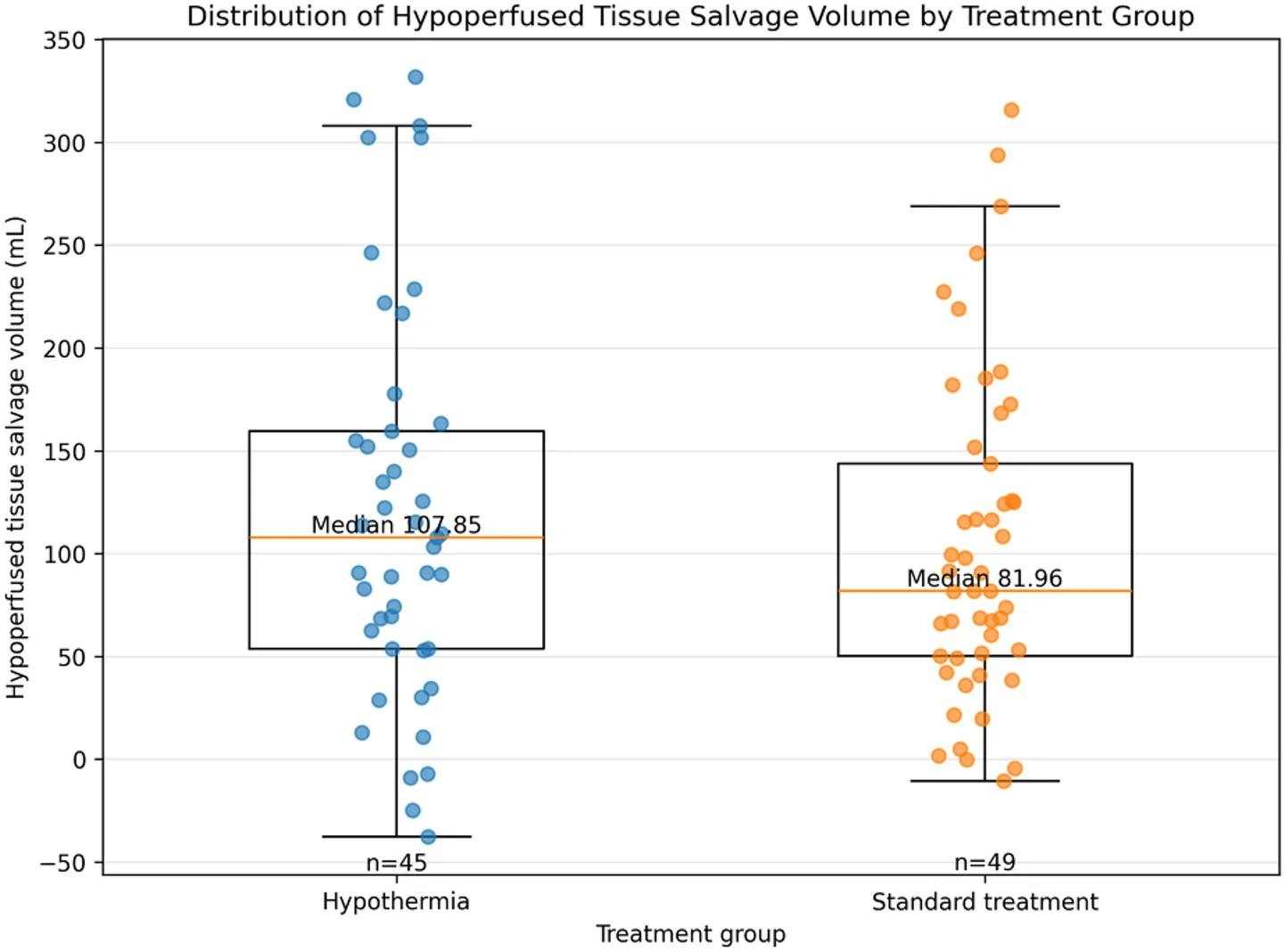
Distribution of hypoperfused tissue salvage volume by treatment group.

Median hypoperfused tissue salvage volume was 107.85 mL (IQR, 53.80–159.64) in the hypothermia group and 81.96 mL (IQR, 50.23–143.80) in the standard-treatment group (Figure 2). Although the point estimate favored the hypothermia group, the between-group difference was not statistically significant in the unadjusted analysis (*p* = 0.384).

In adjusted median regression controlling for age, baseline NIHSS, intravenous thrombolysis, and onset-to-puncture time, selective intra-arterial hypothermia was not significantly associated with hypoperfused tissue salvage volume (adjusted median difference, 21.08 mL; 95% CI, −23.47 to 65.63; *p* = 0.350) (Table 2).

**Table 2:** Imaging and clinical efficacy outcomes.

Outcome	Hypothermia group (*n* = 45)	Standard group (*n* = 49)	Unadjusted *p* value
Primary endpoint: hypoperfused tissue salvage volume^*^, mL	107.85 (53.80–159.64)	81.96 (50.23–143.80)	0.384
Final infarct volume^**^, mL	11.2 (4.0–25.8)	13.1 (2.9–44.4)	0.633
NIHSS improvement from baseline to day 1	2.0 (0.0–4.0)	1.0 (0.0–4.0)	0.347
NIHSS improvement from baseline to day 3	3.0 (1.0–6.0)	2.0 (1.0–5.0)	0.208
NIHSS improvement from baseline to day 7	6.2 ± 5.9	3.8 ± 5.7	0.043
Early neurological improvement at day 7	31 (68.9%)	29 (59.2%)	0.393
90-day mRS 0–1	27 (60.0%)	25 (51.0%)	0.413
90-day mRS 0–2	37 (82.2%)	30 (61.2%)	0.039
90-day mRS 4–6	4 (8.9%)	10 (20.4%)	0.152

The primary endpoint and low-missing continuous variables were displayed after multiple imputation completion for the small number of missing values.

^*^For the primary endpoint, adjusted median regression was performed controlling for age, baseline NIHSS, onset-to-puncture time, and intravenous thrombolysis. The adjusted median difference in hypoperfused tissue salvage volume was 21.08 mL (95% CI, −23.47 to 65.63; *p* = 0.350).

^**^Final infarct volume was calculated based on the MRI performed within 24–48 h. The final infarct volume was calculated using Horos software by manually outlining the infarct borders on each slice, with the total volume obtained by summing the individual slice volumes.

Negative hypoperfused tissue salvage volume occurred in 4 patients (8.9%) in the hypothermia group and 3 patients (6.1%) in the standard-treatment group (*p* = 0.706). In sensitivity analyses, truncating negative values at zero did not materially change the primary-endpoint conclusion (*p* = 0.368). Infarct growth was also analyzed as an alternative imaging metric, and no significant between-group difference was observed (median 5.79 mL vs. 3.60 mL; *p* = 0.820; Supplementary Table S2).

### Secondary efficacy outcomes

Final infarct volume on follow-up MRI was similar between groups: 11.2 mL (IQR, 4.0–25.8) in the hypothermia group versus 13.1 mL (IQR, 2.9–44.4) in the standard-treatment group (*p* = 0.633). NIHSS improvement numerically favored the hypothermia group at day 1 and day 3 and was greater by day 7 (6.2 ± 5.9 vs. 3.8 ± 5.7 points, *p* = 0.043). Early neurological improvement at day 7 occurred in 68.9 and 59.2% of patients, respectively.

**Figure 3:**
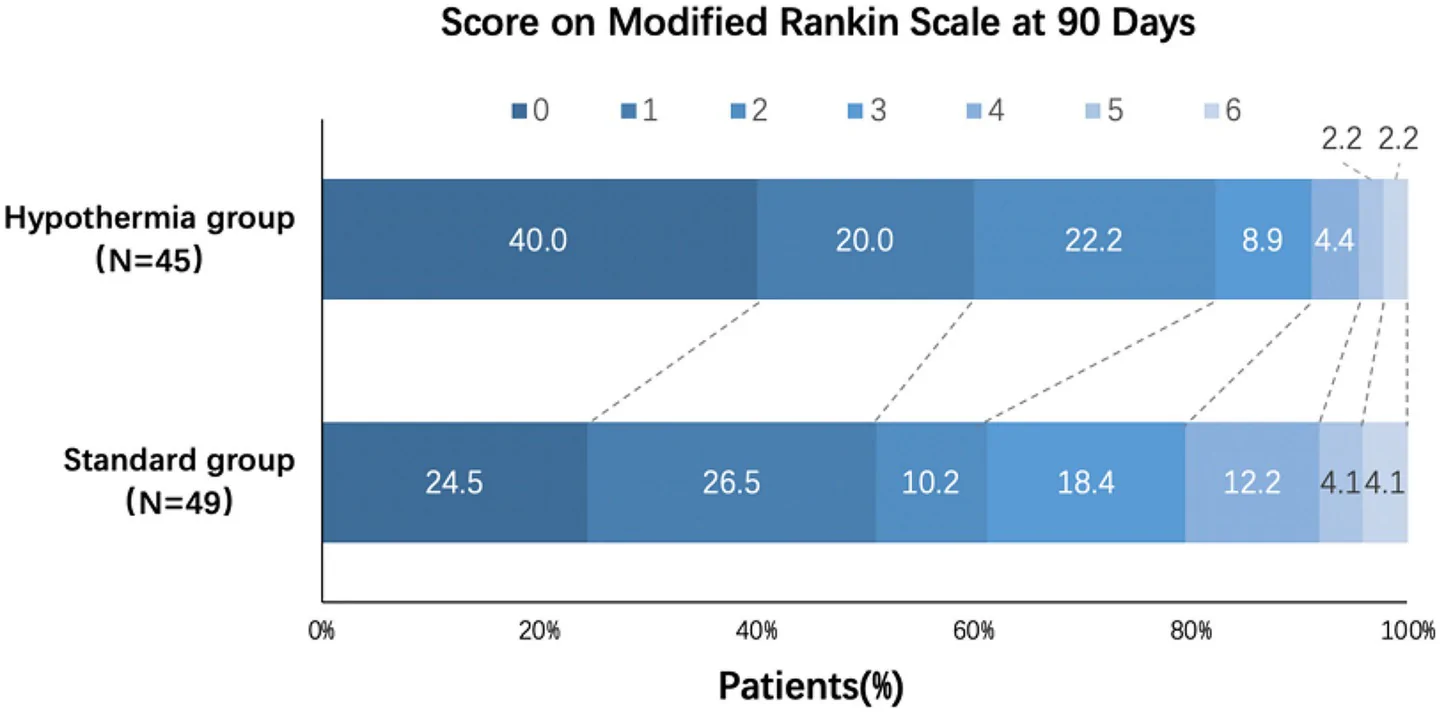
Distribution of 90-day modified Rankin Scale scores.

At 90 days, the mRS distribution numerically favored the hypothermia group, suggesting a shift toward better functional recovery (Figure 3). Functional independence (mRS 0–2) occurred in 37 of 45 patients (82.2%) in the hypothermia group and 30 of 49 patients (61.2%) in the standard-treatment group (*p* = 0.039). Excellent functional outcome (mRS 0–1) occurred in 60.0 and 51.0% of patients, respectively, whereas poor outcome (mRS 4–6) occurred in 8.9 and 20.4%, respectively. In exploratory adjusted analysis, the adjusted risk ratio for 90-day functional independence was 1.41 (95% CI, 1.09–1.81; *p* = 0.008).

### Safety outcomes

**Figure 4:**
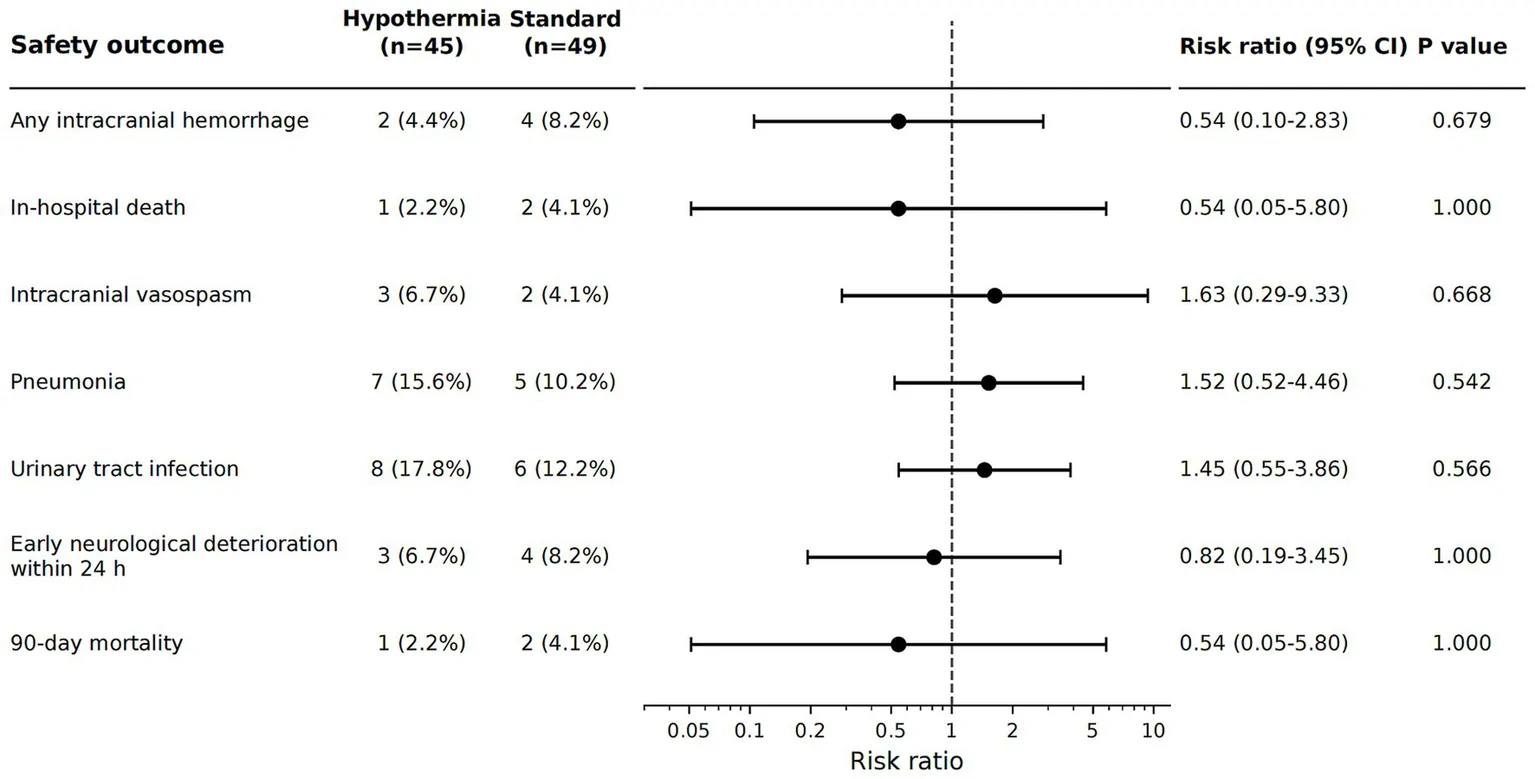
Forest plot of safety outcomes.

Any intracranial hemorrhage on follow-up imaging occurred in 2 of 45 patients (4.4%) in the hypothermia group and 4 of 49 patients (8.2%) in the standard-treatment group. In-hospital death occurred in 1 patient (2.2%) and 2 patients (4.1%), intracranial vasospasm occurred in 3 patients (6.7%) and 2 patients (4.1%), pneumonia occurred in 7 patients (15.6%) and 5 patients (10.2%), urinary tract infection occurred in 8 patients (17.8%) and 6 patients (12.2%), early neurological deterioration within 24 h occurred in 3 patients (6.7%) and 4 patients (8.2%), respectively. Ninety-day mortality occurred in 1 patient (2.2%) in the hypothermia group and 2 patients (4.1%) in the standard-treatment group. No significant between-group differences were observed for any safety outcome (Table 3). Additional peri-procedural laboratory safety parameters are summarized in Supplementary Table S1. Changes in hematologic indices, electrolytes, coagulation parameters, and BNP were generally comparable between the hypothermia and standard-treatment groups. As shown in the forest plot of safety outcomes (Figure 4), there were no statistically significant differences in safety outcomes between the hypothermia and standard treatment groups. The risk ratios for any intracranial hemorrhage, in-hospital death, intracranial vasospasm, pneumonia, urinary tract infection, early neurological deterioration within 24 h, and 90-day mortality all had wide 95% confidence intervals crossing 1.0. These results indicate that selective intra-arterial hypothermia was not associated with an apparent increase in adverse safety events, although the small number of events limits definitive safety conclusions.

**Table 3:** Safety outcomes.

Safety outcome	Hypothermia group (*n* = 45)	Standard group (*n* = 49)	*p* value
Any intracranial, *n*, % hemorrhage, *n*, %	2 (4.4%)	4 (8.2%)	0.679
In-hospital death, *n*, %	1 (2.2%)	2 (4.1%)	1.000
Intracranial vasospasm, *n*, %	3 (6.7%)	2 (4.1%)	0.668
Pneumonia, *n*, %	7 (15.6%)	5 (10.2%)	0.542
Urinary tract infection, *n*, %	8 (17.8%)	6 (12.2%)	0.566
Early neurological deterioration within 24 h^*^, *n*, %	3 (6.7%)	4 (8.2%)	1.000
90-day mortality, *n*, %	1 (2.2%)	2 (4.1%)	1.000

Safety events are shown as *n* (%).

^*^Early neurological deterioration was defined as an increase of ≥4 points in the total NIHSS score from baseline within 24 h after treatment.

## Discussion

In this single-center, nonrandomized comparative cohort, selective intra-arterial hypothermia during mechanical thrombectomy was not associated with a statistically significant increase in hypoperfused tissue salvage volume, although the direction of effect favored hypothermia. This finding suggests that a large tissue-level benefit was not demonstrated in the present sample. At the same time, the result should not be interpreted as evidence against any biological neuroprotective effect. The primary endpoint was designed to reflect the amount of initially threatened tissue that remained noninfarcted after reperfusion, and thus addresses a mechanistically relevant target rather than a purely descriptive imaging change. In addition, exploratory clinical outcomes, including day-7 neurological improvement and 90-day functional independence, numerically favored the hypothermia group, indicating that the treatment signal may not be fully captured by the primary volumetric endpoint alone. The relatively high 90-day functional independence rate, particularly in the standard-treatment group, should be interpreted in the context of the mild baseline stroke severity and high reperfusion rate in this cohort. The median baseline NIHSS score was 7.5 overall and 7.0 in the standard-treatment group, and more than half of the patients had NIHSS scores ≤8. Successful reperfusion was achieved in 94.7% of the overall cohort and 93.9% of the standard-treatment group. Previous studies of low-NIHSS or minor-stroke large-vessel occlusion patients undergoing endovascular treatment, including cohorts with baseline NIHSS ≤5, NIHSS ≤8, or NIHSS <8, have reported 90-day functional independence rates of approximately 63.6–78.3% (15–18). Therefore, the 61.2% functional independence rate in the standard-treatment group appears plausible in the context of this cohort’s lower baseline stroke severity and high reperfusion success. Nevertheless, favorable clinical outcomes may also have been influenced by single-center workflow, patient selection, post-procedural management, and residual confounding. Taken together, selective intra-arterial hypothermia did not show a statistically significant tissue-salvage benefit in this cohort, but it may still have biological and clinical relevance that deserves further evaluation.

To further evaluate the safety profile of the cooling protocol, we additionally analyzed peri-procedural laboratory parameters, including hematologic indices, electrolytes, coagulation parameters, and BNP. Overall, these laboratory changes were generally comparable between the hypothermia and standard-treatment groups and did not suggest an obvious laboratory safety signal related to intra-arterial cooling. Chloride increased modestly more in the hypothermia group, which may be related to the chloride load from intra-arterial infusion of 4 °C normal saline. However, the absolute change was small and was not accompanied by significant between-group differences in sodium or potassium changes, suggesting that this nominal finding did not represent a clinically meaningful electrolyte disturbance. Nevertheless, these laboratory data should be interpreted as supportive safety information and cannot replace standardized core temperature monitoring, continuous cardiac rhythm assessment, or complete serial hemodynamic monitoring.

Pneumonia and urinary tract infection were numerically more frequent in the hypothermia group, although the differences were not statistically significant. Given prior concerns regarding infectious complications with systemic therapeutic hypothermia, particularly pneumonia and sepsis (19), these findings warrant cautious interpretation. Future randomized trials should systematically assess infectious complications when evaluating the safety of selective intra-arterial hypothermia.

Prior studies have consistently suggested that hypothermia is biologically plausible as a neuroprotective strategy because ischemic injury is highly temperature sensitive. Experimental metabolic data indicate that cooling reduces cerebral glucose and oxygen consumption, slows ATP depletion, limits lactate accumulation, and improves energetic recovery during reperfusion (5). In the thrombectomy era, this rationale may be even more important, because successful macrovascular recanalization does not guarantee effective tissue-level reperfusion. Contemporary concepts such as futile reperfusion and no-reflow emphasize that downstream microvascular dysfunction, endothelial injury, blood–brain barrier disruption, oxidative stress, and inflammatory activation can continue to damage tissue even after the occluded artery has been reopened (20). Against this background, selective intra-arterial cooling has a strong theoretical appeal because it is delivered directly into the ischemic vascular territory during the peri-reperfusion period, when these secondary injury processes are most active (21). Consistent with this concept, prior human feasibility work suggests that local cooling of the ipsilateral anterior circulation can be achieved without major systemic temperature disturbance (22). Preclinical studies in nonhuman primates have also suggested that selective cooling may reduce infarct size, preserve white matter integrity, suppress inflammation, and improve functional recovery (23, 24). Early clinical studies likewise point in the same direction, reporting favorable signals in infarct volume, neurological recovery, functional outcome, and biomarker profiles, although these data remain preliminary and do not establish definitive efficacy (9, 25, 26). Against this background, the present neutral primary result is not entirely discordant with earlier work, but rather suggests that any benefit may be more modest, heterogeneous, or more difficult to detect using a single derived imaging measure.

An important novelty of the present study is the choice of hypoperfused tissue salvage volume as the primary endpoint. This metric moves beyond traditional infarct-growth measures and more directly reflects the amount of initially hypoperfused tissue that does not progress to infarction after reperfusion. Conceptually, this endpoint is well aligned with the therapeutic goal of adjunctive neuroprotection during thrombectomy, namely preservation of the ischemic penumbra rather than simple reduction in final lesion expansion. This focus represents a meaningful advance because it attempts to evaluate treatment effect at the tissue level and in a way that is biologically linked to the mechanism of interest (27). In addition, the present analysis highlights a clinically important issue: imaging-based measures of tissue fate and later clinical recovery are not always fully concordant. By showing a neutral primary imaging result alongside more favorable exploratory clinical signals, the study raises the possibility that neuroprotective interventions may influence recovery through pathways not completely captured by gross infarct-volume metrics.

Several considerations may explain the present findings. First, the effect of selective intra-arterial hypothermia may depend not only on whether cooling is delivered, but on the magnitude, timing, and distribution of cooling actually achieved in the downstream ischemic bed. Local brain temperature was not directly measured in this study, and therefore the biological dose of hypothermia remains uncertain, this has also been recognized as a practical limitation in prior translational and clinical work on selective brain cooling (22). Second, the primary endpoint itself has inherent measurement constraints. Hypoperfused tissue salvage volume is a cross-modality derived measure combining baseline perfusion-defined tissue at risk with later MRI-defined infarction. Because these components are obtained with different modalities at different time points, the resulting value is sensitive to imaging timing, reperfusion dynamics, edema evolution, and segmentation variability; negative values may arise for the same reason (27). Third, the apparently more favorable exploratory clinical findings may reflect mechanisms that are not fully expressed as large volumetric differences. Selective cooling may improve the biological quality of reperfusion by preserving microvascular patency, reducing endothelial and blood–brain barrier injury, limiting inflammatory amplification, and protecting vulnerable white matter and peri-infarct tissue. Such effects could plausibly support neurological recovery even if the difference in total salvaged volume remains modest (23). Nevertheless, because treatment allocation was based on operator judgment rather than randomization, residual confounding remains an equally important alternative explanation for the observed clinical signals.

However, several limitations must be acknowledged. The study was single-center, nonrandomized, and modest in size, which limits causal inference, statistical precision, and generalizability. The primary endpoint, while conceptually attractive, is not yet standardized and is vulnerable to cross-modality and timing-related variability. In addition, neither local brain temperature nor downstream tissue-level reperfusion was directly assessed, limiting mechanistic interpretation. Although laboratory safety parameters were analyzed, these data do not replace standardized core temperature monitoring, continuous cardiac rhythm assessment, or complete serial hemodynamic monitoring, which should be incorporated into future prospective trials. Finally, operator-based treatment selection leaves open the possibility of residual confounding in both imaging and clinical outcomes, including the favorable exploratory 90-day functional findings.

Although age was included in the adjusted analysis of the primary endpoint, the hypothermia group was numerically younger, and age-related differences may have influenced the exploratory clinical outcomes. In addition, measured baseline balance, including sex distribution, cannot fully exclude unmeasured factors that may have influenced operator-based treatment selection and outcomes. The applicability of these findings to other populations and practice settings may therefore be limited. Adequately powered randomized controlled trials are needed to further evaluate the efficacy and safety of selective intra-arterial hypothermia during mechanical thrombectomy.

In conclusion, selective intra-arterial hypothermia during mechanical thrombectomy did not produce a statistically significant increase in hypoperfused tissue salvage volume in this cohort, but the direction of effect and the exploratory clinical findings suggest that a biologically meaningful treatment signal cannot be excluded. The study adds to the field by evaluating a more mechanistically relevant tissue endpoint and by highlighting the possibility that neuroprotective benefit may be expressed through improved quality of reperfusion rather than through large shifts in bulk lesion metrics alone. Overall, selective intra-arterial hypothermia remains a biologically credible and clinically testable adjunctive strategy, but larger and more rigorous studies with standardized imaging endpoints, direct temperature verification, and better characterization of downstream reperfusion are required to define its true value.

## Conclusion

Selective intra-arterial hypothermia during mechanical thrombectomy was not associated with a statistically significant improvement in hypoperfused tissue salvage volume, the prespecified primary endpoint. Nominally favorable secondary clinical signals should be interpreted as exploratory. Further adequately powered randomized controlled trials with predefined mechanistic, imaging, and safety endpoints are needed to clarify the efficacy and safety of this approach.

## Data Availability

The raw data supporting the conclusions of this article will be made available by the authors, without undue reservation.
