# Supplementary material for: Hypoperfused tissue salvage after selective intra-arterial hypothermia during mechanical thrombectomy for anterior circulation large-vessel occlusion
Source: Front Neurol. 2026 Aug 12;17:1890819. doi: 10.3389/fneur.2026.1890819 (PMC13506298; doi:10.3389/fneur.2026.1890819)
Supplement: Supplementary file 1 [file Table_1.DOCX]

# Table S1. Periprocedural laboratory safety parameters before and after treatment

| **Variable** | **Hypothermia pre** | **Hypothermia post** | **Hypothermia change** | **Standard pre** | **Standard post** | **Standard change** | **P for change** |
| --- | --- | --- | --- | --- | --- | --- | --- |
| CRP | 1.89 (0.63-3.62) | 2.23 (1.30-4.85) | 0.37 (0.00-1.24) | 1.81 (0.50-4.74) | 2.90 (0.85-11.96) | 0.99 (-0.06-5.91) | 0.439 |
| WBC | 7.94 (6.32-10.13) | 9.18 (7.10-12.30) | 1.27 (0.08-2.67) | 7.85 (5.76-9.82) | 9.43 (8.00-11.31) | 1.65 (-0.08-4.08) | 0.325 |
| RBC | 4.84 (4.46-5.06) | 4.35 (3.96-4.62) | -0.38 (-0.59--0.22) | 4.58 (4.11-4.98) | 4.18 (3.76-4.56) | -0.41 (-0.59--0.23) | 0.976 |
| Hemoglobin | 147.00 (139.00-152.00) | 133.00 (120.00-146.00) | -12.00 (-19.00--4.00) | 139.00 (131.00-148.00) | 129.00 (116.00-139.00) | -10.00 (-17.00--5.00) | 0.702 |
| Hematocrit | 42.90 (40.60-44.30) | 39.00 (35.90-41.90) | -3.40 (-5.60--1.50) | 42.20 (39.10-44.60) | 39.30 (35.60-41.70) | -3.00 (-5.30--1.90) | 0.853 |
| Potassium | 3.75 (3.48-4.02) | 3.92 (3.54-4.10) | 0.10 (-0.20-0.36) | 3.95 (3.76-4.08) | 3.97 (3.64-4.21) | 0.01 (-0.25-0.33) | 0.508 |
| Sodium | 140.00 (139.00-142.00) | 141.00 (139.00-143.00) | 1.00 (-1.00-3.00) | 140.00 (139.00-141.00) | 141.00 (139.00-144.00) | 2.00 (0.00-3.00) | 0.222 |
| Chloride | 105.00 (103.00-107.00) | 108.00 (105.00-110.00) | 2.00 (0.00-5.00) | 106.00 (103.00-108.00) | 106.00 (104.00-109.00) | 0.00 (-1.00-4.00) | 0.04 |
| PT | 12.20 (11.70-12.80) | 13.00 (12.40-14.00) | 0.90 (0.30-1.50) | 12.40 (12.10-13.20) | 13.30 (12.90-13.80) | 0.50 (0.20-1.20) | 0.218 |
| APTT | 28.00 (25.10-31.00) | 31.50 (29.00-33.30) | 2.00 (0.30-6.20) | 29.40 (25.10-33.10) | 30.20 (27.80-32.40) | 1.00 (-1.20-4.70) | 0.12 |
| D-dimer | 0.65 (0.47-0.92) | 1.32 (0.74-2.32) | 0.57 (0.11-1.49) | 0.78 (0.55-1.06) | 1.95 (1.09-3.92) | 0.82 (0.26-2.42) | 0.376 |
| BNP | 32.40 (15.62-63.62) | 35.45 (24.70-113.95) | 9.05 (-0.53-26.08) | 34.30 (14.20-121.40) | 61.80 (34.60-221.40) | 19.30 (4.80-47.00) | 0.085 |

*Values are median (interquartile range). Change was calculated as postoperative minus preoperative value. P values compare change values between groups using the Mann-Whitney U test. Analyses were restricted to patients with available paired pre- and post-procedural values. The chloride change reached nominal statistical significance; this result should be interpreted cautiously in the context of multiple laboratory comparisons and the modest absolute difference.*
