# Supplementary material for: Hypoperfused tissue salvage after selective intra-arterial hypothermia during mechanical thrombectomy for anterior circulation large-vessel occlusion
Source: Front Neurol. 2026 Aug 12;17:1890819. doi: 10.3389/fneur.2026.1890819 (PMC13506298; doi:10.3389/fneur.2026.1890819)
Supplement: Supplementary file 2 [file Table_2.DOCX]

# Table S2. Sensitivity Analyses for the Primary Imaging Endpoint

| **Analysis** | **Hypothermia group**  **(n=45)** | **Standard group**  **(n=49)** | **P value** |
| --- | --- | --- | --- |
| Negative hypoperfused tissue salvage volume, n (%) | 4 (8.9%) | 3 (6.1%) | 0.706 |
| Hypoperfused tissue salvage volume after truncating negative values at zero, median (IQR), mL | 107.85 (53.80–159.64) | 81.96 (50.23–143.80) | 0.368 |
| Infarct growth, median (IQR), mL | 5.79 (0.70–16.17) | 3.60 (0.32–14.20) | 0.820 |

*Hypoperfused tissue salvage volume was defined as baseline hypoperfusion volume on CT perfusion minus final infarct volume on follow-up MRI. Values are shown as n (%) or median (IQR), as appropriate. The P value for negative salvage volume was calculated using Fisher’s exact test; P values for continuous variables were calculated using the Mann-Whitney U test.*
